# Neuromuscular choristoma: a rare cause of congenital non-progressive lower limb amyotrophy

**DOI:** 10.1590/0004-282X-ANP-2020-0370

**Published:** 2021-05-09

**Authors:** Roberta Ismael Lacerda MACHADO, José Marcos Vieira de ALBURQUERQUE, Paulo Victor Sgobbi de SOUZA, Laís Uyeda AIVAZOGLOU, Bruno de Mattos Lombardi BADIA, Stéphanie Yuri Torres OGATA, Igor Braga FARIAS, André Yui AIHARA, Artur da Rocha Corrêa FERNANDES, Wladimir Bocca Vieira de Rezende PINTO, Acary Souza Bulle OLIVEIRA

**Affiliations:** 1 Universidade Federal de São Paulo, Departamento de Neurologia e Neurocirurgia, Divisão de Doenças Neuromusculares, São Paulo SP, Brazil. Universidade Federal de São Paulo Universidade Federal de São Paulo Departamento de Neurologia e Neurocirurgia Divisão de Doenças Neuromusculares São Paulo SP Brazil; 2 Universidade Federal de São Paulo, Departamento de Diagnóstico por Imagem, Divisão Osteomuscular, São Paulo SP, Brazil. Universidade Federal de São Paulo Universidade Federal de São Paulo Departamento de Diagnóstico por Imagem Divisão Osteomuscular São Paulo SP Brazil; 3 Diagnósticos da América S/A, DASA Group, São Paulo SP, Brazil. Diagnósticos da América S/A DASA Group São Paulo SP Brazil

**Figure 1.**
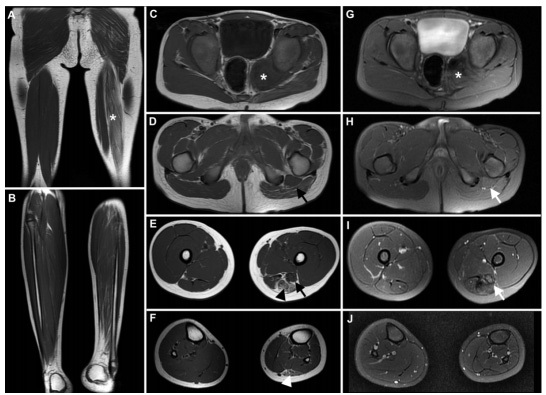
Muscle magnetic resonance (MR) imaging studies of the lower limbs. Coronal T1-weighted MR image of the thighs shows fatty substitution of the long head of the biceps femoris on the left thigh (asterisk) (A). Coronal T1-weighted MR image of the lower legs shows substantial asymmetry with diffuse hypotrophy of the left lower leg muscles in comparison to the contralateral unaffected side (B). Axial T1-weighted images of the pelvis (C), thighs (D and E) and lower legs (F) show a mass in the pelvis (C; asterisk) that merges with the left sciatic nerve (D and E; black arrows), which shows fusiform enlargement, with homogeneous intermediate signal intensity, similar to the adjacent muscle bellies. The fatty replacement of the long head of the biceps femoris (E; black arrowhead) and of the lateral head of the left gastrocnemius (F; white arrowhead) are shown. Axial T2-weighted fat-saturated images show intermediate signal intensity of the pelvic mass (G; asterisk) and the enlarged sciatic nerve (H and I; white arrows). Homogeneous Intermediate signal in T2- and T1-weighted images similar to adjacent muscle bellies is highly suggestive of neuromuscular choristoma (asterisk and arrows).

**Figure 2.**
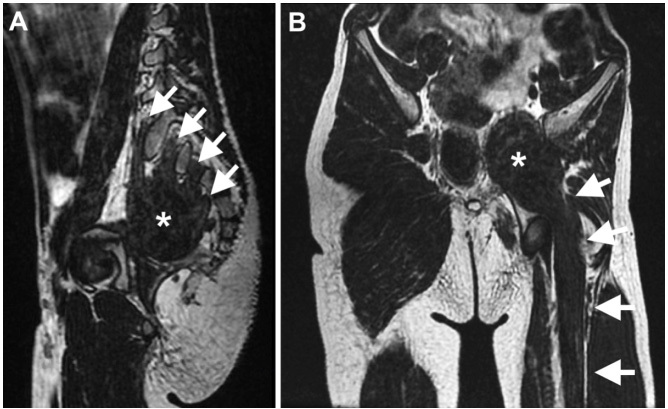
Magnetic resonance imaging studies of the pelvis. Oblique sagittal reformatted T2-weighted MR image of the pelvis shows contribution of the left L5 to S3 nerve roots (white arrows) to form the pelvic mass (asterisk) (A). Oblique coronal reformatted T2-weighted MR image of the pelvis shows continuity of the pelvic mass (asterisk) with the enlarged sciatic nerve (arrows) and the similarity of its signal intensity with the adjacent muscle bellies (B).

A 7-year-old boy presented with non-progressive amyotrophy of the left lower limb since birth. Examination disclosed isolated amyotrophy of the left lower limb. Sphincteric disturbances and fasciculations were not present. Muscle and pelvic magnetic resonance imaging (Figures 1 and 2) disclosed findings highly suggestive of neuromuscular choristoma (NMC) of the left sciatic nerve.

NMCs of the sciatic nerves, formerly known as neuromuscular hamartomas, are rare tumors with mature skeletal muscle and neural elements^1^^,^^2^. Typical imaging features are important for differential diagnosis, especially in cases with congenital long-standing involvement and monomelic amyotrophy, avoiding unnecessary biopsy and the risk of developing aggressive fibromatosis^1^^,^^2^.
