# Erratum

**DOI:** 10.1590/0004-282X-ANP-2020-0370er

**Published:** 2022-08-08

 In the article “Neuromuscular choristoma: a rare cause of congenital non-progressive lower limb amyotrophy”, with DOI code number https://doi.org/10.1590/0004-282X-ANP-2020-0370, published in the Arq Neuropsiquiatr. 2021;79(5):465-6, page 465: 

**Where it was written:**

Roberta Ismael Lacerda MACHADO^1^, José Marcos Vieira de ALBURQUERQUE FILHO^1^, Paulo Victor Sgobbi de SOUZA^1^, Laís Uyeda AIVAZOGLOU^2,3^, Bruno de Mattos Lombardi BADIA^1^, Stéphanie Yuri Torres OGATA^3^, Igor Braga FARIAS^1^, André Yui AIHARA^2,3^, Artur da Rocha Corrêa FERNANDES^2^, Wladimir Bocca Vieira de Rezende PINTO^1^, Acary Souza Bulle OLIVEIRA^1^

**Should read:**

Roberta Ismael Lacerda MACHADO^1^, José Marcos Vieira de ALBUQUERQUE FILHO^1^, Paulo Victor Sgobbi de SOUZA^1^, Laís Uyeda AIVAZOGLOU^2,3^, Bruno de Mattos Lombardi BADIA^1^, Stéphanie Yuri Torres OGATA^3^, Igor Braga FARIAS^1^, André Yui AIHARA^2,3^, Artur da Rocha Corrêa FERNANDES^2^, Wladimir Bocca Vieira de Rezende PINTO^1^, Acary Souza Bulle OLIVEIRA^1^

